# The Experiences of Family Dyads During the Gender Identity Transition Process of Children and Adolescents

**DOI:** 10.1111/scs.70291

**Published:** 2026-07-07

**Authors:** Augusto Krindges, Guilherme da Silva Biasus, Carolina Andrade Costa, Tania Christiane Ferreira Bispo, Anderson Reis de Sousa, Jeferson Santos Araújo

**Affiliations:** ^1^ Federal University of Fronteira Sul (UFFS) Chapecó Brazil; ^2^ Federal University of Bahia (UFBA) Salvador Brazil; ^3^ University of the State of Bahia—UNEB Salvador Brazil; ^4^ Postgraduate Program in Nursing and Health, School of Nursing Federal University of Bahia Salvador Brazil; ^5^ Postgraduate Program in Nursing, School of Nursing Federal University of Fronteira Sul Chapecó Brazil

**Keywords:** adolescents, children, gender identity, nursing care, transgender people

## Abstract

**Objective:**

This study aims to analyse the transition process experienced by mother/father dyads when they recognise their children's gender dysphoria.

**Methodology:**

The study employed a qualitative approach, based on interpretative description and the theoretical support of the transitions theory, in which 10 dyads participated. The interviews were conducted using a semi‐structured script, recorded, transcribed, and subjected to reflective thematic analysis.

**Results:**

Revealed a central theme: transitioning together. This entails the facilitating and inhibiting factors that best describe the experiences of these dyads.

**Conclusion:**

The theory is a resource for healthcare professionals to recognise phenomena that help or hinder the transition processes of children and adolescents.

## 1 Introduction

Gender dysphoria (GD) is a form of discomfort or a disorder that a person develops in relation to their birth sex and how it is perceived and expressed in their behaviours throughout life, characterized by suffering related to the gender assigned at birth [1]. Although its manifestation can occur in adults or during puberty, its first characteristics usually emerge in childhood [2].

Researchers [1] highlight that quantifying this data is a challenge due to the lack of specific studies that can accurately reflect the reality of this population, since current data only focuses on individuals who wish to undergo hormone therapy or who have already undergone surgical sex reassignment procedures. According to [3], gender dysphoria is more prevalent in boys (2.2%) than in girls (0.5%), although gender incongruence is more frequent among girls during childhood.

A study [4] highlights that several transgender people, at some point in their childhood and adolescence, experienced some persistent discomfort regarding their biological sex, identifying with a gender opposite to their birth sex. During childhood and adolescence, identifying as male or female, or occasionally with a different designation, becomes a dilemma in the construction of gender identity that can result in the development of significant symptoms of psychological stress [4], motivating several individuals to seek measures to alter their body characteristics (through gender affirmation surgeries and hormonal therapies) in order to adjust to the gender with which they identify [5, 6].

Dissatisfaction with one's own body and the associated dissociative disorders and traumas during childhood was the theme of a longitudinal study conducted with 118 Italian individuals with GD, which showed a high prevalence of depressive episodes throughout life (45.8%), suicide attempts (21.2%), and childhood traumas (45.8%), generating strong psychological suffering which was only mitigated by treatment aimed at sexual reassignment surgery [7].

Researchers have reported that parents are an important support network for children and adolescents when they are deciding whether or not to pursue sexual reassignment or gender transition [8].

A study conducted in Boston, Massachusetts, involving 180 transgender youths aged 12 to 29, 106 trans men and 74 trans women, found that many participants experienced declines in mental health, including depression (50.6%), anxiety (26.7%), suicidal ideation (31.1%), suicide attempts (17.2%), and non‐suicidal self‐injury (16.7%). When compared with cisgender individuals, these rates were significantly higher across all outcomes assessed [9].

Universal access to sexual healthcare services is one of the challenging goals of the United Nations and its member states to be met by the year 2030 in the achievement of the Sustainable Development Goals [10]. The health of children and adolescents with GD during their gender identity transition can be an integral part of achieving this goal, as researchers [5] point out that an increasing number of children, adolescents, and their families seek paediatric healthcare services for GD‐related care, highlighting the need for guidelines to determine how to best provide effective and comprehensive care.

The specific literature on GD highlights that a child's gender identity transition affects all family members, as the parents responsible for the child or adolescent may feel guilt and have doubts regarding the decisions made and support provided during the gender transition [11]. Therefore, parents need to receive and provide support when facing the challenges that go beyond the biological issues of their child's body, and which include bullying, discrimination, lack of adequate healthcare, disapproval from the family and community, and the lack of social understanding regarding gender transition [12]. However, while studies have documented parental experiences regarding gender transition in children and adolescents [13, 14, 15], the primary gap in the literature lies in the lack of geographical diversity. As highlighted in the critical review by [14], research remains predominantly concentrated in the USA, Canada, Europe, and Oceania.

### 1.1 Theoretical Background

The Transitions Theory is based on the notion of transitioning from an unstable condition to a stable one and aims to describe, interpret, understand, and explain phenomena related to role changes, which produce behavioural and social transformations of the self. The theory is structured on the nature of transitions, which are conditioning factors for the transition and response patterns [16].

Its nature is related to the patterns and properties that develop as a result of transitions generally, that is, it explains the types of transition an individual undergoes during the course of their development. Its conditioning factors highlight the situations that work as facilitators and inhibitors linked to the individual's personal, community, and social issues during the transition. Response patterns are subdivided into progress indicators and outcome indicators, which characterize the development of the transition generally. In this setting, therapeutic interventions can be developed for a healthy transition [16].

## 2 Objective

To analyse the transition process experienced by mother/father dyads when they recognise their children's gender dysphoria and attempt to adjust to their new supporting roles.

## 3 Method

### 3.1 Type of Study

This is a qualitative study using interpretative description [17] and Meleis's transitions theory [16], whose development complied with the Consolidated Criteria for Reporting Qualitative Research (COREQ) guidelines [18].

Interpretive description is an inductive theoretical framework suitable for studying applied clinical problems. Due to its flexibility, its application is useful when interpreting data, as it generates rich results at both the descriptive (superficial) and interpretive (deeper) levels, which are able to report structured patterns in a coherent story that is intertwined with the cultural context, literature, and theory [18].

### 3.2 Inclusion or Exclusion Criteria

To be included in the study, participants were required to: (a) be parents of a child or adolescent aged 10 to 18 who reported early feelings of dysphoria, characterised by a strong preference for clothing, play roles, and toys typically associated with the other gender during childhood that persisted into adolescence, in accordance with DSM‐5 criteria [19]; (b) be the parents of a child or adolescent diagnosed with gender dysphoria; (c) be the parents of a child or adolescent undergoing gender identity transition. The exclusion criteria applied were: (a) self‐declared cognitive impairment which prevented them from participating in the interview and answering the study questions; (b) refusal by one of the members of the father/mother dyad to participate in the study.

### 3.3 Study Setting and Recruitment

We employed purposive sampling and conducted recruitment using the Snowball technique, where an individual has the opportunity to refer another to participate in the study and so on, establishing links between members of a given group [20]. The first and last authors conducted the recruitment by initially analysing information provided by parents of children and adolescents with GD who had an early initiation of gender transition according to the DSM‐5 [19], who participated in a specific Non‐Governmental Organization (NGO) that advocates for the causes of the LGBTQIAPN+ population (Lesbian, Gay, Bisexual, Transvestite and Transsexual, Queer, Intersex, Asexual, Pansexual, and Non‐Binary), located in a city in the countryside of the state of Santa Catarina, Brazil. Information such as the age and diagnosis of the children was verified to ensure eligibility criteria.

**FIGURE 1:**
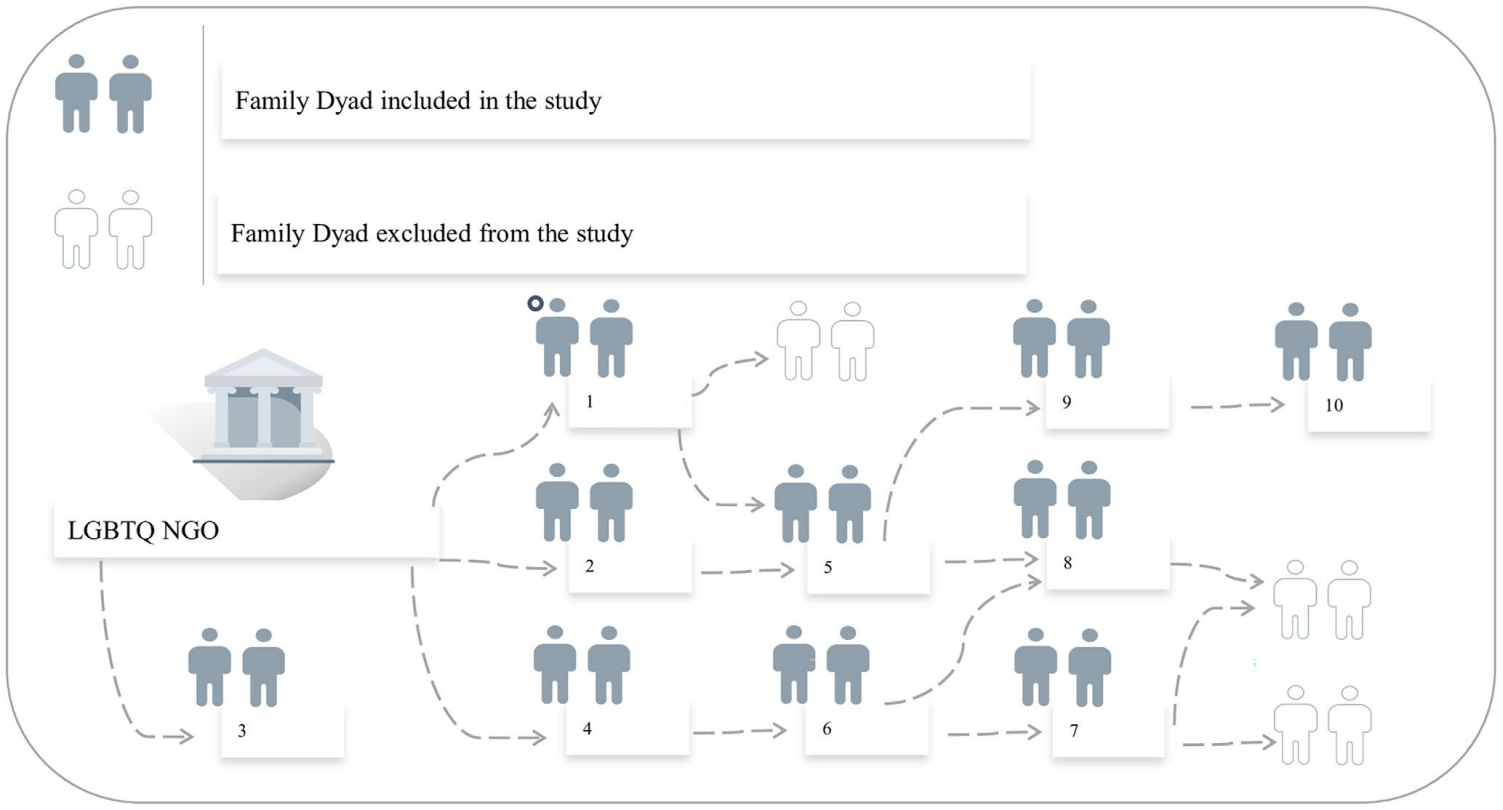
Visual representation of the methodological process. *Source*: Prepared by the researchers.

The researchers first contacted four dyads, providing information regarding the study. They all agreed to participate and provided their contact details to schedule an interview date. During the initial contact, three dyads referred other dyads, which then referred more dyads, as shown in Figure 1.

In total, 14 eligible dyads were contacted. Four refused to participate in the study as they did not feel comfortable recounting their children's gender transition experiences. As a result, 10 dyads were interviewed in person.

### 3.4 Data Collection

From June 2022 to February 2023, the first author conducted two interviews (3 months apart) individually with each dyad. The researcher had previous experience in collecting qualitative data and was not involved in monitoring the participants' gender transition.

The interviews were conducted at the times and places where the dyads stated they felt most comfortable. They all chose to participate in the interviews at their homes. During the interview meetings, the dyads were offered the option of being interviewed together or separately, since the nature of the research theme is sensitive and communication about family relationships in the face of gender identity transition is still taboo in Brazilian culture, and it is recognised that some people may not feel comfortable being accompanied when talking about the subject. However, all the dyads decided to be interviewed together, suggesting that they felt comfortable talking freely in the presence of their spouses.

The semi‐structured interviews were conducted using a script developed based on existing literature [1, 18], as well as the clinical expertise of the research team.

The guiding question used to start the interviews was: Could you tell me about your experience of your child's gender identity transition, as if it were a book? Based on the answer to this question, other questions were asked and adapted for each dyad to deepen the data collection, such as:

- How do you deal with your child's gender identity transition issues? Why?
- What have you been doing that could possibly facilitate your child's gender identity transition? Why?
- What have you been doing that could possibly inhibit your child's gender identity transition? Why?

The interviews lasted a minimum of 27 min and a maximum of 45 min (36 min on average), and were digitally recorded and transcribed in full. Data collection concluded when the data provided sufficient depth and diversity to address the research question. Consistent with recent methodological critiques of ‘saturation’ as information redundancy [21], we prioritized the richness of the dataset over a specific point of code recurrence.

### 3.5 Data Analysis

Reflective thematic analysis, structured in six stages, was used to analyse the research data [22]: (I) Data familiarization. Repeated readings of the transcribed texts were performed; (II) Identification of the initial codes. For this stage, we used the MaxQDA qualitative data analysis software, which allowed us to read the data in depth and explore the content in search of answers to the study's objective, integrating the information from the field diary notes and participant notes from the perspective of two different levels of connected analysis: (1) representation in quantitative terms (frequency) of the coded segments and qualitative terms (the meaning they conveyed), (2) exploration of the similarities and differences present in the experiences provided by the participants; (III) construction of themes. Once the initial codes had been identified, it was possible to organize the data set in search of themes, which were then submitted to hermeneutic analysis, an analytical method that allows the researcher to report on phenomena (experiences) and interpret their meanings in an understandable context [23]. This stage was conducted in three phases: in the first phase, a general analysis was performed on the quotes that support the initial codes (initial comprehension), which allowed the researchers to achieve a basic understanding of the essential meaning of the conditioning factors of transitions generally. In the second phase, a structured analysis was carried out, objectively refining the data into units of meaning, which highlight the overlapping meanings of passages narrated by the participants in a theoretical setting. The units of meaning were grouped together, and inductively, then sub‐themes were created (for instance: the secure attachment bond, tolerance, the flow of time, among others). The sub‐themes favoured a focused understanding of the gender transition phenomenon and were used to create the theme. (IV) Theme review. The theme was submitted to an initial evaluation, after which it was read by a third researcher and all the quotes, codes, and sub‐themes were discussed with the research team for critical reflection. (V) Defining and naming the theme. At this stage, the theme was continuously examined and validated in the context of the “initial understanding” of the hermeneutic analysis, in order to represent the participants' message as closely as possible to their lived reality. This was followed by the third phase of hermeneutic analysis, in which the sub‐themes and themes were synthesized and named. (VI) Report writing. The results were constructed and then interpreted from the perspective of the transitions theory [16], thus providing a broad understanding of the phenomenon.

### 3.6 Rigour and Reflexivity

Key elements such as credibility, transferability, dependability, and confirmability were adopted to establish the rigour and reflexivity of the study [24]. Each stage of the research and the methodological decisions were detailed. The interview script was validated by two researchers external to the research group while the constraints, sub‐themes, and main theme were refined during multiple rounds of debate between peers. All the researchers have previous experience in qualitative research: the first author is a nurse, who is a master's Nursing student, specialising in primary care with a focus on the LGBT population; the second author is an undergraduate Nursing student, trained in the development of qualitative methods; and the other authors are PhDs with extensive experience in the development of research with transition generally and healthcare themes centred on children and adolescents.

To reduce researcher bias, the entire research team had no previous relationship with the participants or the NGO, and to ensure research rigour, the COREQ checklist was followed, in addition to the audited trail to illustrate the excerpts narrated by the participants.

### 3.7 Ethical Considerations

The study was approved by the institutional research ethics review board with approval number 5.481.769. Written informed consent was granted by all the dyads prior to the interviews and the participants' anonymity was ensured by replacing their real names with an alphanumeric code consisting of the letter “D”, followed by the number of the sequential order in which the dyads were included in the research.

## 4 Results

### 4.1 Participants

A total of 10 father/mother dyads participated in the study. All were heterosexual and their religious belief was Christianity, with a majority of ages over 45, white skin race and ethnicity, and elementary school education. Among their children, three were born female, aged from 16 to 18, and seven were born male, aged from 10 to 17. More details are shown in Table 1.

**TABLE 1:** Characteristics of the family dyads (*n* = 10).

Variable	Father		Mother	
	*N*	%	*N*	%
*Age in years*				
25–34	2	20	4	40
35–44	2	20	2	20
> 45	6	60	4	40
*Self‐declared race*				
White	9	90	8	80
Black	1	10	2	20
*Education status*				
Elementary school	5	50	5	50
Middle school	1	10	1	10
Undergraduate	2	20	4	40
Postgraduate	2	20		
*Occupation*				
Nurse	1	10	1	10
Teacher	2	20	3	30
Other^a^	7	70	6	60

^a^ Entrepreneur; housekeeper; painter; public servant.

**FIGURE 2:**
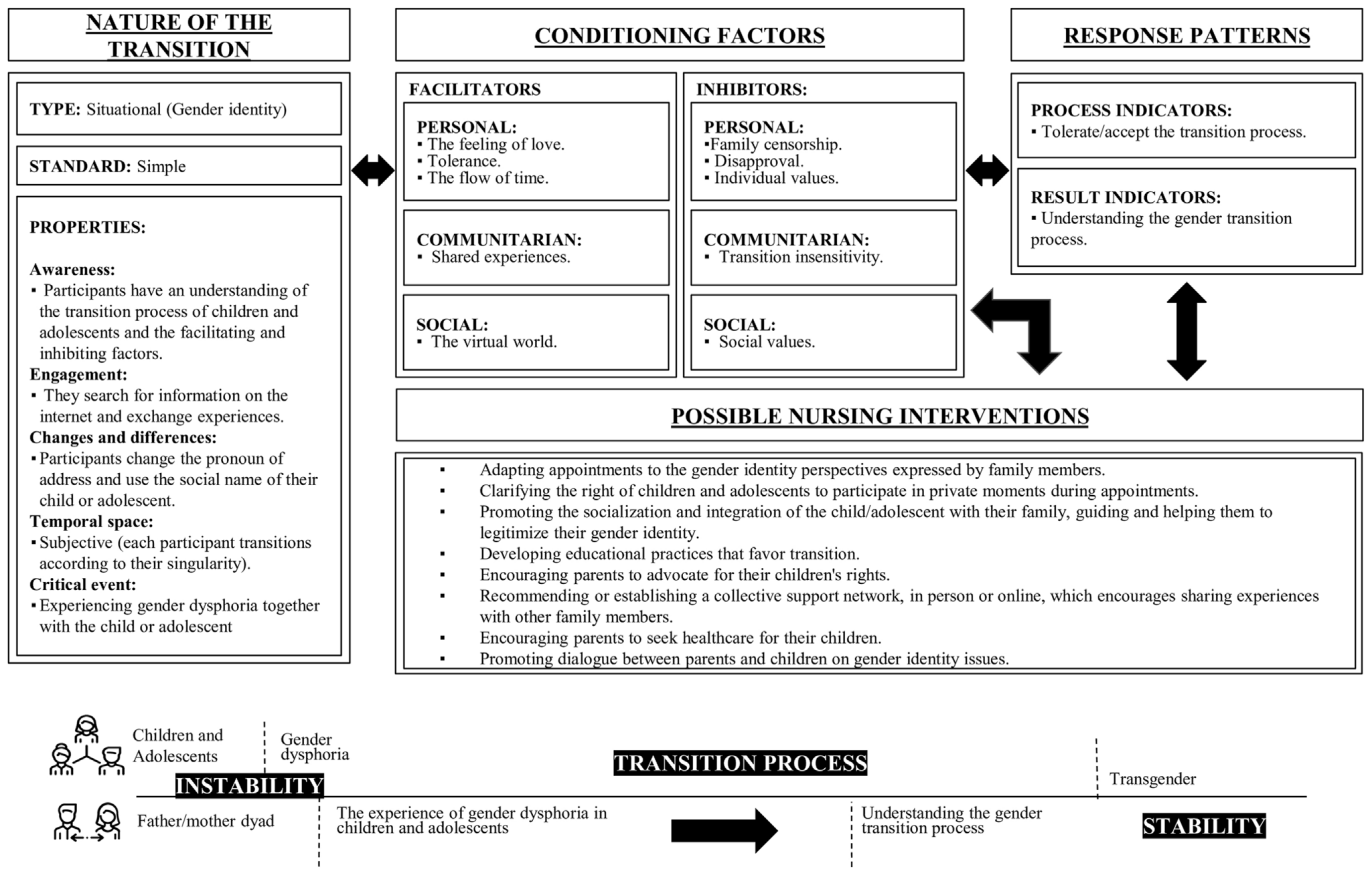
Data synthesis based on the transitions theory. *Source*: Prepared by the researchers.

Based on the transitions theory, the data analysis enabled interpreting the gender transition process, from the parents' perspective, in relation to their new roles in supporting their children with gender dysphoria. Figure 2 has been organized to better elucidate the theoretical process developed in the research.

We have identified 10 sub‐themes: (1) Secure attachment bond; (2) Tolerance; (3) The flow of time; (4) Sharing experiences; (5) Virtual media; (6) Censorship; (7) Disapproval; (8) Self‐judgement; (9) Transition insensitivity; and (10) Social values. The sub‐themes provide support for interpreting the conditioning factors that facilitate and inhibit the gender transition, thus favouring, through reflection and synthesis, the creation of a central theme entitled: Transitioning together.

### 4.2 Main Theme: Transitioning Together

#### 4.2.1 Facilitating Conditioning Factors for the Transition

Regarding the personal factors that facilitate gender transition, the dyads highlighted the development of secure attachment bonds with their child as a form of coping with the challenges. Upon discovering GD and experiencing the transition process, the participants were faced with a combination of sensations and thoughts, as exemplified in the following excerpts:

A child is for life! A child is a child! So nothing has changed for me, it helps me to support her. (D1)

When he told me, the shock was great, I'm not going to say it wasn't, the shock was huge, I cried a lot in church, then came home, hugged him, and said: “He's my son, right! He's my blood, I'm not going to do anything, I'll just help him. I fight for him, I even stopped going to relatives and places that don't accept him. If he's no good, I'm no good either, I love him!” (D4)

The dyads noticed that their child's gender transition is a complex process, which requires attention and behavioural change. Therefore, in an effort to facilitate this personal process, they chose to tolerate the adversities they experienced, as evidenced in the following reports:

I know everything we have to go through, so I'm going to go through it, because I have to accept my daughter just the way she is, because she's my daughter. I didn't want this for her, but I'm going to help her however I can. (D1)

If it's his will, I'll always be here, my love won't change, I'll respect him and support him in whatever he needs, regardless of what he's becoming, I think that's the best way to help him in this process, regardless of whether I agree or not. (D3)

The flow of time proved to be a facilitator of personal transition for the dyads, as it strengthened concepts that enabled the promotion of healthy experiences that facilitate gender transition, as exemplified in the following excerpts:

[…] It's a process for him and for me too, and like every process, it takes time. Time is the best medicine. (D2)

In fact, it was a shock. She was a girl, and we had been calling her one gender, female, for 15 years, and out of the blue she wanted to impose herself and for us to refer to her as “he”. So I said: “Look, unfortunately it takes time, it's not overnight that your head turns around and you switch genders.” I said it would take a while and said we should choose a nickname so we wouldn't have to call her by that name yet. And that's how we call her at home, we call her “baby”. (D3)

From a communitarian perspective, sharing experiences with other family members enables strengthening and helping the children's gender transition process, as a support network is established to exchange knowledge, as evidenced by the statements below:

*And today society is much more open, things are more in the spotlight, we can count on the experience of other people to understand the processes our children go through*. (D6)

*Gus is transgender, and I have a son, Ramon, who is gay! So homosexuality, gay people, and transgender people are a very common topic here at home, so we already know what these transition issues are like and when we can count on the support of people who have already been through these experiences, we feel more empowered to help them* (referring to the son). (D7)

Acting as a personal transition facilitator, the information propagated by virtual media was highlighted by the dyads as an important communication channel in their search to understand their children's rights and the processes they face when transitioning, as described in the following reports:

She started with a phase where she wanted to change her name at school, saw it on social media, and showed it to me. And then she showed up with a printed law saying that trans people had this form of support, that people had this authorization to be called by their social name so that they would feel less uncomfortable at school. I signed the paper for him, but it all started after she saw it on the internet and showed it to me. (D3)

And I went there, I researched whether he could use his social name at school. So, I know because some of the things we're discovering are really only available on the internet, and this network allows us to communicate with other trans people who are going through this. (D7)

#### 4.2.2 Inhibiting Conditioning Factors for the Transition

Insecurity and fear of supporting their children in their decisions to assume their gender identity is a personal inhibiting factor in the transition and is related to the fact that other family members adopt a censorious stance, as can be seen in the following excerpts:

I kept it a secret for a while, because I was afraid he would be kicked out of the house, because of the examples we had seen in other families, and then I couldn't stand it any longer and I told my husband. Even though I was afraid, I told him. (D4)

When the family wants to be cruel, it really is! You suffer the most violence at home, when you repress your child and they don't see you as their support, they feel insecure and don't tell you anything (emotional voice, showing a sense of relief)! So I think that, depending on the family, it can be one of the worst places, you can be repressed if you try to help […]. (D6)

For some dyads, upon discovering that their children are undergoing gender transition, the feeling of disapproval was expressed as a personal inhibitor of the process. For the time being, ignoring or denying the possibility of the existence of a transgender child was reported as an issue that protects them socially, as can be seen in the following excerpts:

I couldn't accept the idea that it was my girl, I couldn't accept the fact that I called her as him. I know it's my own prejudice and it doesn't help, but for me it's her and that's that! I don't want her to go through what we see on TV, involving death, prejudice, abuse, and prostitution. (D2)

I ignored it! I told myself it was a lie, and I never brought it up again! Do you know why? Because society sees everything about them as bad, so I ignored the fact that my son was like that. (D4)

Another personal inhibiting factor in the gender transition highlighted by the dyads concerns the self‐critical stance on their past behaviour and self‐judgement due to the fact that they didn't realize what was happening earlier, which can negatively interfere in their child's transition process, as evidenced in the following excerpts:

In fact, I kept asking myself: “How have I never noticed this before?” Am I a bad mother? Am I a mother who didn't pay enough attention? If I had realized this before, I could have helped my child go through all this! (D2)

Regardless of sex or color, I think we've changed a lot, but how often do I catch myself being prejudiced? Sometimes I don't mean to be. Because it's something that's ingrained, like structural racism. It's that thing inside your head, only it's been regarded as natural. (D6)

The dyads reported that insensitivity to recognizing and respecting children/adolescents in the gender transition phase is a community constraint that inhibits their process:

You have to be careful who you hang around with, you see, there are a lot of people who are evil, who pick on you, abuse you, and kill you, who make fun of you (practice bullying), that's what prejudice is, which gets in the way a lot during this process and ends up really messing with us. Not everyone respects or is prepared to help, you have to be sensitive enough to put yourself in the other person's shoes. (D1)

After he really discovered himself as a man, due to people not understanding what he was going through, he decided to change schools, because at the old school he was already known and suffered discrimination, which didn't help at all, and at the new school he could be happy being whoever he wanted to be, because there he would be a new student. (D7)

Social values were highlighted by the dyads as social elements that inhibit their children's gender transition process, on the one hand presenting themselves as a metric that children and adolescents are expected to reproduce and, on the other, as a confusing factor in communication that hinders freedom of gender expression in the social environment:

We sent out two birthday invitations, one to the family with his registered name, and the other with the name he's called, because not everyone agrees with his process. But at the party, the photographer said “Oh, let's take some pictures of her” (calling him by the registered name) and one of the friends said “Who is that person?” Because nobody knew that name, for them it's him, so society judges him constantly and limits him to going through all this. (D6)

Because people expect something from you, but our son doesn't want to give it. And you're not obliged to give people what they expect. The problem is that we hear a lot of things that only make this process more difficult, and we end up being emotionally attacked. (D9)

## 5 Discussion

When a child or adolescent with GD transitions through their gender identity, the entire family transitions together, since directly or indirectly the transition promotes physical, behavioural, and social changes. It is within their families that individuals experience primary socialization, learn norms, personal values, and how to relate to the world. These acquisitions are essential for identity formation.

According to the context being researched, the experience of GD during the process of gender identity transition starts with a trigger. For children, adolescents, and the father/mother dyad, this experience causes instability and leads them to gender transition in search of stability (for children and adolescents, the challenge is to affirm themselves as transgender to society, and for the dyad, to build an understanding of the transition process). The nature of gender transition is that it is situational, as there are changes in the social roles of all those involved, and that it is simple, as it is a single transition on which we have focused our analysis.

The dyads experienced the child's gender transition in a subjective way, as each one transitioned according to their own singularities. To this end, it became clear that the process hinges on the temporal context in which the social actors find themselves, as transitional time is fluid. Therefore, it depends on how engaged each dyad is in the process of striving to find a balance with the children's/adolescents' transitional phase.

The balance for conducting a healthy gender transition is related to secure attachment and family support. Researchers [25] point out that a lack of family support can have a negative impact on the gender transition, leading to denial, discrimination, suicidal ideation, non‐adherence to healthcare services, behavioural and psychological problems, among other problems.

For the dyads, time is an essential factor in finding this balance, as experiencing their child's identity changes entails elements that both facilitate and hinder the process. For instance, as in the results of this research, studies [26] state that the feeling of love for their children provides a secure attachment bond that enables dyads to tolerate the tensions that emerge with the new gender identity. The existence of a sense of belonging and family bonds would be associated with an increase in the confidence, self‐esteem, and well‐being of dysphoric children/adolescents. As well as aspects related to lasting family bonds, acceptance and respect for gender identity would be key components in dealing with the transition [27]. According to the transitions theory [16], environmental conditions and family and community support (experiences shared with other parents) help interaction and observation of the multiple possibilities for understanding the phenomenon.

For the dyads, the adoption of a new gender identity for their children creates instability in family relationships, which leads to disapproving behaviour, censorship, and uncertainty regarding the future, since each family member deals with the gender transitional process according to their individual and social values that regulate their culture. Thus, many choose to neglect their child's gender transition, repress their feelings, and self‐blame their past behaviours in the search for explanatory causes as to why their children are undergoing gender transition, which inhibits the process. Although some dyads display tolerant behaviour, this does not necessarily represent a movement towards acceptance, which requires more time for understanding the phenomenon.

Studies such as those by [25] point out that disapproval, lack of support and balance in family relationships favour a vulnerability process, as the stigmas created influence parents' self‐perception of their children, hindering them from experiencing a healthy gender transition. Researchers [12] presented an evidence synthesis that reinforces the results of this study; from 32 studies analysed, they highlighted that parents' stigma regarding their children's gender identity results in negative experiences for their mental health.

The experiences, when shared between the dyads, favour family support, strengthen engagement, and the exchange of information on how to deal with gender transition. Whether on social networks or in other media, information works as an aid for understanding, including the existing legal processes that allow for a safer gender transition, as well as demonstrating other experiences similar to those that the dyads and their children are facing or may face in the future. According to a study [28], support through online connections with other people in the gender transition process and also with virtual communities can reduce isolation, depression, and loneliness. However, as with all virtual media, it is worth noting that these groups are susceptible to cyberbullying, defined as any type of violence and form of aggression practiced in the virtual environment, which can lead to negative experiences, complications in the transition process, and psychological suffering for individuals [29]. Thus, as Meleis' theory of transitions (2010) implies, it is vital that the entire transition process occurs with the support of family, friends (whether virtual or not), the various communities, and healthcare professionals, so that the entire process works in a healthy and safe way, with as little psychological damage as possible for adolescents and children going through these changes.

Research in this area remains scarce in Brazil, a country characterized by binary family structures and entrenched hegemonic masculinities. Additionally, Brazil has held the position of the world's leader in murders of transgender people for 18 consecutive years [30].

## 6 Study Limitations

In general, a limitation of this research is the heterogeneity of the participants, as no family members other than the father/mother dyad took part. Their participation could have provided complementary perspectives on the gender transition process, thereby strengthening data triangulation. It is suggested that future research should explore the experiences of other family members, healthcare professionals, and children, in order to describe and interpret common and diverse aspects related to the gender transition process. It is also recommended to carry out longitudinal studies to explore the gender transition process at different stages of life.

## 7 Contributions to the Nursing Field

This study contributes to the enrichment of the literature in Paediatric and Hebeatric Nursing, since, by considering the experiences of the dyads who live with their children's gender transition on a daily basis, it provides greater visibility to their facilitators and inhibitors that interfere in the gender transition process, which influence adherence to healthcare and, therefore, should be considered by nurses in their interventions. Possible nursing interventions are represented in Figure 2 and can be applied in clinical practice to promote health.

This study contributes to the literature in Paediatric and Adolescent Nursing by considering the experiences of dyads navigating their children's daily transitions. It provides greater visibility to the facilitators and inhibitors that influence the gender transition process and, consequently, healthcare adherence. These factors should be integrated into nursing interventions, ensuring that consultations are adapted to the gender identity perspectives expressed by family members. Furthermore, the potential nursing interventions illustrated in Figure 2 can be applied in clinical practice to promote health and facilitate healthy transitions.

## 8 Concluding Remarks

The dyadic relationship experienced by this study's participants was based on the theoretical framework of transitions, which helped to identify as the main results the factors that facilitate the gender identity transition process of children and adolescents with GD, such as the development of secure attachment bonds with their child, tolerance, the understanding that time favours adjustment to the flow of the transition, sharing experiences with other families, information provided by the media, and also the conditioning factors that inhibit the gender transition process, such as censorship of information about the child's transition to other family members, disapproval, individual and social values, as well as a certain insensitivity towards the transition. Based on the results, it is hoped that possible nursing interventions can be provided to the dyads, which is why future studies are advised.

## Author Contributions

Conception, J.S.A.; Methodology, J.S.A.; Formal analysis, J.S.A.; Research, J.S.A., G.S.B. and A.K.; Resource collection, J.S.A.; Writing – original draft preparation, J.S.A., G.S.B., A.K., C.A.C., T.C.F.B., A.R.S.; Writing – proofreading and editing, J.S.A., G.S.B. and A.K. Visualization, J.S.A.; Supervision, J.S.A., and C.A.C.; Funding acquisition, J.S.A.

## Funding

This work was supported by Federal University of Fronteira Sul—Brasil [UFFS]—Public Notice 73/GR/UFFS/2023/Funding Code: PES‐2023‐0301.

## Ethics Statement

The Ethics Committee for Research with Human Beings of the Federal University of Fronteira Sul approved our interviews (approval number 5.481.769) on June 2022. The interviewees provided written consent for review and signed the term before the interviews were conducted.

## Consent

The interviewees provided written consent for review and signed the terms before starting the interviews.

## Conflicts of Interest

The authors declare no conflicts of interest.

## Data Availability

The data that support the findings of this study are available on request from the corresponding author. The data are not publicly available due to privacy or ethical restrictions.
